# Drainage Tube Placement May Not Be Necessary During Endoscopic Thyroidectomy Bilateral Areola Approach: A Preliminary Report

**DOI:** 10.3389/fsurg.2022.860130

**Published:** 2022-03-10

**Authors:** Yukai Chen, Chengchen Wang, Binglong Bai, Mao Ye, Junjie Ma, Jingying Zhang, Zhiyu Li

**Affiliations:** ^1^Department of Thyroid Surgery, Second Affiliated Hospital of Zhejiang University, School of Medicine, Hangzhou, China; ^2^Department of General Surgery, International Medical Center of Second Affiliated Hospital of Zhejiang University, School of Medicine, Hangzhou, China

**Keywords:** drainage, endoscopic thyroidectomy, areola approach, papillary thyroid carcinoma, complications, hematoma

## Abstract

**Background:**

The endoscopic thyroidectomy bilateral areola approach (ETBAA) improved cosmetic outcomes significantly and is now widely applied. The usage of drainage tubes is controversial in conventional open thyroidectomy (COT), but studies about drainage placement decisions during ETBAA are still limited. This study aimed to determine the feasibility of having no drainage tube applied during ETBAA on patients with papillary thyroid carcinoma.

**Methods:**

The clinical data of patients undergoing ETBAA from July 2018 to May 2021 was retrospectively collected. The patients were divided into two groups based on drain placement: no-drain and drain. The two groups were matched at a ratio of 1:1. Fifty-five patients from each group were finally included. Postoperative complications and follow-up data were compared between the two groups.

**Results:**

No significant difference was observed between the two groups in the incidence of postoperative complications, including hemorrhage, surgical site infection, and subcutaneous seroma. Compared with the drain group, the operation time of the no-drain group was significantly shorter [(107.75 ± 24.59) min vs. (119.91 ± 34.05) min, *P* < 0.05]. The total and postoperative hospital stay was significantly shorter in the no-drain group [(2.40 ± 0.71) days vs. (4.78 ± 1.33) days, *P* < 0.001, (2.04 ± 0.19) days vs. (2.15 ± 0.36) days, *P* < 0.05], and the costs of surgical consumables were also significantly lower [(6,820.83 ± 164.29) CNY vs. (7,494.13 ± 216.7) CNY, *P* < 0.05]. The postoperative pain score of the no-drain group was significantly lower than the drain group [(1.58 ± 0.63) vs. (1.89 ± 0.76), *P* < 0.05].

**Conclusions:**

No drainage applied during ETBAA on papillary thyroid carcinoma is safe and feasible. This practice does not increase the risk of postoperative complications, but it does shorten the operation time and hospital stay, as well as reduce medical costs. Furthermore, it alleviates the suffering of patients.

## Introduction

The incidence rate of thyroid cancer has been steadily increasing for some years and has a dominant incidence in females (1, 2). Surgery is the primary treatment for thyroid cancer. Traditionally, thyroidectomy is performed via a transcervical incision, which leaves a prominent scar on the anterior neck. Therefore, various surgical techniques have emerged to improve the cosmetic outcomes and life quality of thyroidectomy patients. Multiple surgical approaches, such as bilateral axillo-breast (3, 4), transaxillary (5), areola (6), and transoral (7, 8), have been developed since the introduction of endoscopic thyroidectomy. The endoscopic thyroidectomy bilateral areola approach (ETBAA) is the most commonly used.

To avoid a noticeable neck scar, the incision must be made at a distant location, and surgeons must separate the anterior neck region and dissect the skin flap during ETBAA to create the space. This would result in a large dead space in the surgical site, which could lead to complications such as hemorrhage, infection, and seroma. Thus, one drainage tube is conventionally left across the suprasternal fossa via the right areola incision after ETBAA in China. However, an increasing number of patients complained about the inconvenience and surgical site pain caused by the drainage. Accumulating studies suggest that strict and effective intraoperative hemostasis is essential in preventing postoperative hemorrhage and that routine drainage tube insertion in COT is unnecessary (9–13). Furthermore, the use of drainage prolongs the hospital stay and increases the incidence of surgical site infection and hematoma (9, 10, 13). However, it still lacks evidence on whether patients who underwent ETBAA could benefit from the insertion of postoperative drainage. No drain application has been conducted in our center for years in COT, and it has also been conducted in all ETBAA patients since August 2020. Therefore, this study was designed to assess the safety and feasibility of no drainage applied in ETBBA. We present the following article in accordance with the STROBE guidelines.

## Methods

### Study Design

This was a retrospective case-controlled clinical study. The study was approved by the Ethics Committee of the Second Affiliated Hospital, Zhejiang University School of Medicine (Ethic approval No. 2021-0583). Patients were enrolled from a cohort of 212 continuous patients who underwent thyroidectomy via ETBAA at our facility between August 2018 and May 2021. Since August 2020, we have completely changed our drain strategy from one routine drain placement to no drain. Inclusion criteria were as follows: (1) female patients under the age of 55; (2) the surgical approach is ETBAA; (3) no signs of lateral cervical lymph node or distant metastasis before the operation; (4) postoperative pathology confirmed the diagnosis of papillary thyroid carcinoma. Exclusion criteria included: (1) previous history of thyroid surgery; (2) a surgical approach of COT, endoscopic thyroidectomy via oral vestibular approach, or axillary approach; (3) patients undergoing lateral cervical lymph node dissection; (4) a history of hyperthyroidism, hypertension, diabetes, coagulation dysfunction. It is worth noting that the endoscopic thyroidectomy was mainly applied in young female patients with cosmetic demands, and male patients who underwent ETBAA were excluded due to a lack of numbers. Under these criteria, 57 patients were enrolled in the no-drain group from August 2020 to May 2021, and 93 patients from August 2018 to July 2020 were enrolled in the drain group. Making use of a 1:1 case-control match in SPSS 26.0, 55 patients in the experimental group were matched with 55 patients in the drain group. The matching criteria were thyroidectomy range and maximum tumor size.

### Surgical Procedures

ETBAA was performed using a method similar to that described by Wang et al. (14). To reduce intraoperative hemorrhage, a special visual separation bar and trocar, as previously described, were used (15). In the drain group, one drainage tube was routinely placed in the thyroid fossa and emerged through an incision in the areola, whereas no drainage was used in the no-drain group. Postoperative complications were appropriately managed. As soon as postoperative hemorrhage was detected, emergency operations were launched. For patients diagnosed with seroma, percutaneous aspirations were repeated until the seroma disappeared. And intravenous antibiotics were used to treat surgical site infections. Patients were followed up for 1 month after surgery to evaluate short-term postoperative drainage-related complications.

### Outcomes

The primary outcomes were postoperative drainage-related complications including hemorrhage, hematoma, subcutaneous seroma, and surgical site infection, all of which were evaluated by the same medical team. Hemorrhage and hematoma were defined as postoperative bleeding that manifested as acute swelling of the anterior cervical sites, necessitating a subsequent operation (15, 16). Subcutaneous seroma was defined as chronic subcutaneous swelling and fluctuation that required aspiration. Surgical site infection was defined as a postoperative surgical site abscess that required antibiotic treatment (16, 17).

Secondary outcomes included pain evaluation, hospital stay, operation time, surgical consumable costs, and inflammation-related clinical characteristics. The Visual Analog Pain Scale/Score (VAS) was used to assess pain on a daily basis until the patient was discharged. Patients who needed postoperative oral pain medication were also documented. The hospital stay included both the total hospital stay and the postoperative hospital stay. The highest postoperative body temperature and perioperative blood test results, including levels of C-reaction protein (CRP), white blood count (WBC), neutrophil ratio, and neutrophil count, were used to assess inflammation.

### Statistical Analysis

The data was analyzed using SPSS 26.0 software (SPSS Inc., Chicago, IL, USA). Continuous variables were expressed as mean ± standard deviation and were analyzed by the *t*-test. Categorical variables were analyzed by the Pearson chi-square test or Fisher's exact test. The differences with a *p* value <0.05 were considered statistically significant.

## Results

**Figure 1:**
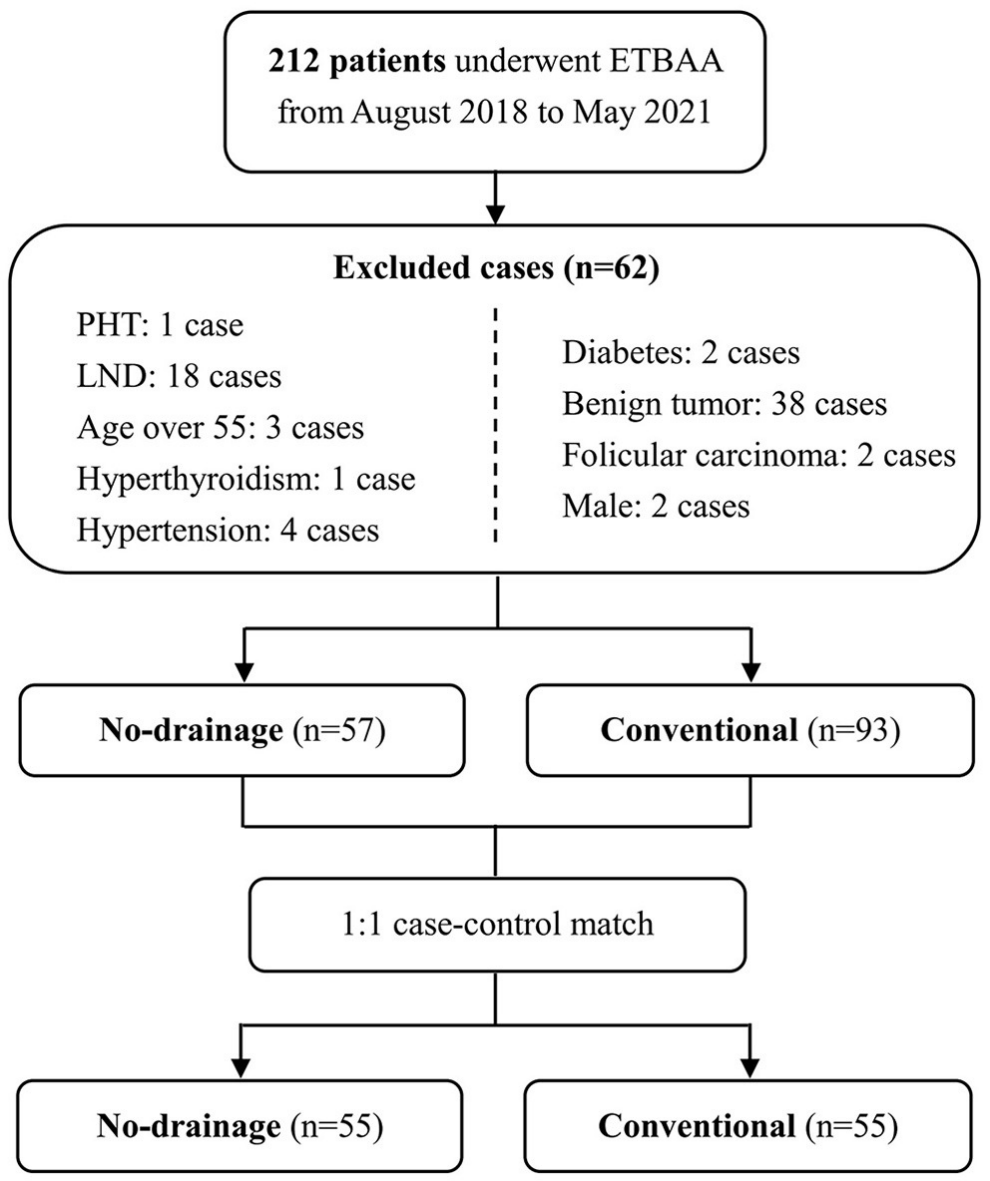
Flow diagram of patient selection and matching. *PHT*, past history of thyroidectomy; *LND*, lateral neck dissection.

The clinicopathological characteristics of the two groups were comparable, with no significant differences (*P* > 0.05) (Table 1 and Figure 1). All ETBAA were successfully performed with no conversion to open surgery. There were no cases of postoperative hemorrhage or hematoma. Three patients in the no-drain group and one patient in the drain group experienced subcutaneous seroma. One patient underwent surgical site infection 1 week after surgery and recovered well after receiving intravenous antibiotics. There was no statistically significant difference in the rate of postoperative complications between the two groups. The detailed data is listed in Table 2.

**Table 1:** Clinicopathological characteristics of patients apply no drainage vs. patients routinely apply one drainage tube.

**Characteristics**	**No-drain group (*n* = 55)**	**Conventional group (*n* = 55)**	***t* value**	***p* value**
Age (mean ± SD, years)	33.76 ± 7.18	32.24 ± 7.24	1.111	0.269
BMI (mean ± SD, kg/m2)	22.36 ± 3.24	21.53 ± 3.86	1.228	0.222
Maximum tumor diameter (mean ± SD, cm)	0.74 ± 0.44	0.73 ± 0.48	0.187	0.852
Operation [*n* (%)]				
Unilateral thyroidectomy	43 (78.18%)	43 (78.18%)	–	–
Bilateral thyroidectomy	12 (21.82%)	12 (21.82%)	–	–

*BMI, Body Mass Index*.

**Table 2:** Patients with drainage-related complications.

**Case**	**Complication**	**Group**	**Occurrence** **day after surgery**	**Volume of aspirations (ml)**	**Number of aspirations**
1	Subcutaneous seroma	No-drain	21	5	1
2	Subcutaneous seroma	No-drain	7	10	1
3	Subcutaneous seroma	No-drain	7	15	1
4	Subcutaneous seroma	Conventional	10	10	1
5	Surgical site infection	Conventional	7	–	–

Table 3 shows the secondary outcomes in the two groups. In the drain group, the postoperative drainage time was 1.16 ± 0.46 days and the postoperative total drainage volume was 65.67 ± 39.26 ml. The operation time was significantly reduced in the no-drain group (107.75 ± 24.59 min) compared with the drain group (119.91 ± 34.05 min) (*P* = 0.034). The total hospital stay and postoperative hospital stay of the no-drain group were also significantly shorter than the drain group [(2.40 ± 0.71) days vs. (4.78 ± 1.33) days, respectively, *P* < 0.001, (2.04 ± 0.19) days vs. (2.15 ± 0.36) days, respectively, *P* = 0.048]. And the no-drain group had significantly lower surgical consumable costs than the drain group [(6,820.83 ± 164.29) CNY vs. (7,494.13 ± 216.7) CNY, *P* = 0.015]. Furthermore, the VAS scores at 8 a.m. the next day after surgery were significantly lower than those of the drain group [(1.58 ± 0.63) vs. (1.89 ± 0.76), respectively, *P* = 0.022]. Nonetheless, there were no differences in VAS scores on discharge day between the two groups [(1.40 ± 0.63) vs. (1.16 ± 0.81), *P* = 0.090]. Five patients in the drain group took temporary pain medication after surgery, while the number in the no-drain group was one. However, no significant differences were found (*P* = 0.216). There were no differences inpostoperative inflammation-related data between no-drain and drain groups, including the highest postoperative body temperature [(37.52 ± 0.29) °C vs. (37.48 ± 0.25) °C, *P* = 0.779], postoperative CRP [(11.36 ± 6.68) mg/l vs. (9.45 ± 4.87) mg/l, *P* = 0.111], postoperative WBC count [(8.23 ± 2.40) vs. (8.00 ± 2.11), *P* = 0.614], postoperative neutrophil ratio [(6.56 ± 2.24) vs. (6.13 ± 2.06), *P* = 0.324], and postoperative neutrophil count [(77.15 ± 13.15) % vs. (75.5 ± 7.57) %, *P* = 0.432]. All data is described in Table 3.

**Table 3:** Perioperative clinical data of patients apply no drainage vs. patients routinely apply one drainage tube.

**Variables**	**No-drain group (*n* = 55)**	**Conventional group (*n* = 55)**	***t* value**	***p* value**
Operation time (mean ± SD, min)	107.75 ± 24.59	119.91 ± 34.05	−2.148	0.034
Total hospital stay (mean ± SD, day)	2.4 ± 0.71	4.78 ± 1.33	−11.724	0.000
Postoperative hospital stay (mean ± SD, day)	2.04 ± 0.19	2.15 ± 0.36	−2.008	0.048
Costs of surgical consumables (mean ± SD, CNY)	6,820.83 ± 164.29	7,494.13 ± 216.7	−2.476	0.015
VAS pain score (mean ± SD)	1.58 ± 0.63	1.89 ± 0.76	−2.320	0.022
8 a.m. the next day after surgery				
Discharge day	1.40 ± 0.63	1.16 ± 0.81	1.710	0.090
Cases using oral pain medications (*n*)	1	5	–	0.206*^#^*
Maximum postoperative body Temperature (mean ± SD,°C)	37.52 ± 0.29	37.48 ± 0.25	0.779	0.438
CRP (mean ± SD, mg/l)				
Before surgery	3.09 ± 2.77	3.2 ± 4.68	−0.129	0.898
One day after surgery	11.36 ± 6.68	9.45 ± 4.87	1.613	0.111
White Blood Count (WBC)				
Before surgery	5.99 ± 1.59	5.84 ± 1.3	0.546	0.586
One day after surgery	8.23 ± 2.40	8.00 ± 2.11	0.505	0.614
Neutrophil count (NEUT^#^)				
Before surgery	3.66 ± 1.08	3.41 ± 1.08	1.201	0.107
One day after surgery	6.56 ± 2.24	6.13 ± 2.06	0.992	0.324
Neutrophil ratio (NEUT%, %)				
Before surgery	59.79 ± 11.11	57.71 ± 8.87	1.085	0.280
One day after surgery	77.15 ± 13.15	75.5 ± 7.57	0.789	0.432

*VAS, Visual Analog Scale/Score; CNY, China yuan.*

# *Fisher's exact test*.

## Discussion

As a consequence of the yearly increasing incidence and the female predominance of thyroid disease (1, 2), cosmetic demands are growingly concerned. The postoperative “suicide” scars on the anterior neck impose psychological burdens on patients, particularly young females, which provides prospects for scarless surgery. The first endoscopic parathyroidectomy performed by Gagner in 1996 opened a new era of endoscopic thyroid surgery (18). Numerous approaches, such as the axillo-breast, areola, axillary, and oral vestibular approach (3–8), have been developed since then. ETBBA, developed by M Ohgami (6) is the most common approach at present. The surgical technology was mature, and studies revealed a similar complication rate and lymph node dissection thoroughness when compared to COT, but with a longer operation time. The disadvantages of ETBAA are acceptable, while the therapeutic effects and safety are granted (14, 19). In general, ETBAA is effective and safe for thyroid disease treatment and has a broad application in young female patients.

Postoperative hemorrhage is the major severe complication of thyroid surgery that usually occurs within 24 h after surgery (15), which could be fatal considering the rich blood supply and trachea-ahead location of the thyroid. Postoperative hemorrhage after ETBBA is rare, with an incidence rate of 0.32–0.724% (14, 15), which is similar to COT (0.43–4.39%) (20, 21). The drainage tube was found to be ineffective in preventing postoperative bleeding. Numerous studies, including a large-scale retrospective study (12), a randomized clinical trial (11), and meta-analysis (9, 10, 13), have proved drainage should not be routinely employed in COT. Drainage does not reduce the risk of complications like hemorrhage, recurrent laryngeal nerve palsy, and seroma, but increases the incidence of surgical site infection and hematoma. The necessity of routine drainage has been widely challenged in COT, especially in the United States.

Unlike COT, there has been little research into drainage placement decisions during endoscopic thyroidectomy. Endoscopic thyroidectomy, as opposed to COT, requires broad flap separation of the anterior neck and chest area, which may increase bleeding. Considering this, postoperative drainage after ETBAA is still applied routinely (14, 15, 22, 23). However, a retrospective study in our institution reported a small number of patients after ETBAA suffered from hemorrhage and hematoma after the drainage tubes were removed (0.103%, 2/1932) (15), suggesting that removal of the drainage tubes may cause extra tissue laceration and may lead to severe outcomes. With the advancement of surgical techniques and equipment, as well as an improving understanding of postoperative hemorrhage, surgeons challenged the routine placement of drainage after ETBAA. Novel energy equipment like bipolar scalpels and ultrasonic scalpels makes the closure of small blood and lymphatic vessels more reliable, lowering the incidence of postoperative hemorrhage (24, 25). The common application of novel energy equipment during ETBAA makes it possible not to use drains. And the utilization of the special visual separation bar and trocar can help better control intraoperative hemorrhage (15). Chen et al. changed the position of the drainage tube in order to achieve better cosmetic outcomes (26). In this study, we abandoned the use of drainage directly, reporting no severe postoperative complications. No cases of hemorrhage or hematoma in either group were observed. Notably, no routine drainage application during ETBBA should be done with caution and under certain conditions. Patients with male sex, advanced age, benign thyroid goiter, hypertension, diabetes, coagulation dysfunction, history of thyroid surgery, and lateral lymph node dissection were excluded from this study, as these are risk factors for postoperative hemorrhage (15, 27, 28). For these high-risk patients, the decision to not apply drainage must be made with greater caution, and more studies in these populations are needed. Based on our experience, a drainage tube should be placed under these circumstances. Firstly, excessive exudation is detected after strict intraoperative hemostasis. Secondly, massive bleeding occurred during the operation, especially in the period of building working space. Thirdly, thoracic duct injury is suspected. Besides risk factors, careful intraoperative hemostasis is considered the most important way of preventing hemorrhage (11). Thus, the surgeons should be experienced and familiar with ETBAA to perform this practice. For beginners, the application of drainage is still recommended. Perioperative management and intense monitoring in the first postoperative 24 h are vital as well. Swelling on the chest wall and neck, which mostly appears within 24 h after operation, is the most typical manifestation of hematoma after ETBAA (15). It is critical to recognize hematoma as soon as possible to avoid fatal bleeding. In addition, postoperative management of cough, vomiting, hypertension, and strenuous neck activity was crucial for decreasing hemorrhage incidence.

Aside from hemorrhage, subcutaneous seroma and surgical site infection are two major complications associated with drainage usage. A meta-analysis revealed no differences in terms of seroma formation between endoscopic thyroidectomy and COT (3.9 vs. 2.5%, *P* > 0.05) (29). The comprehensive prevalence of seroma formation after endoscopic thyroidectomy was 2.9–4.7% (17, 29, 30). We reported comparable rates of subcutaneous seroma of 5.5% (3/55) in the no-drain group and 1.8% (1/55) in the drain group. All four patients underwent percutaneous aspiration once with a 20 ml injection syringe. The average aspiration volume is 10.00 ± 4.08 ml. No recurrence was reported, and all four patients were satisfied. There was no significant difference in subcutaneous seroma incidence between the two groups (*P* = 0.618). Previous literature reported aspiration volumes ranging from 6 to 120 ml with 1 to 7 times aspiration (30), suggesting the subcutaneous seroma in our study is relatively mild. Concerning postoperative infections, only one patient in the drain group developed surgical site infection 7 days after surgery and recovered well with intravenous antibiotics treatment. No case of infection was observed in the no-drain group. And no significant difference was found between the two groups regarding the highest postoperative body temperature, postoperative CRP, WBC, neutrophil counts, or neutrophil ratio. The application of drainage in ETBAA may increase the incidence of infection in the same way that it does in COT (11, 13), while evidence from a larger sample size is required.

The postoperative drainage time for patients with drainage insertion after ETBAA was 1.16 ± 0.46 days. Most patients removed drainage tubes on the first postoperative day and were discharged 2 days after surgery, which was significantly shorter than previous literature. Wang et al. (14) and Chen et al. (26) reported the drainage time with 2.7 ± 0.6 days and 4.3 ± 0.9 days, respectively, and the hospital stay with 6.4 ± 1.2 days and 6.4 ± 0.8 days, respectively. Previous studies revealed that drain placement is associated with more extended hospital stay in COT (9–12), and the application of negative pressure drainage is an important reason that interferes with lymph tube sealing and increases drainage volume (31). In the present study, with no drainage applied, the total hospital stay significantly reduced from 4.78 ± 1.33 days to 2.40 ± 0.71 days (*P* < 0.001), and postoperative hospital stay decreased considerably from 2.15 ± 0.36 days to 2.04 ± 0.19 days (*P* = 0.048). The operation time was also significantly shortened from 119.91 ± 34.05 min to 107.75 ± 24.59 min (*P* = 0.034). Moreover, there is considerable cost-reduction for not using drains from 7,494.13 ± 216.7 CNY to 6,820.83 ± 164.29 CNY (*P* = 0.015). The reduction of the average hospital stay, operation time, and medical costs improves the efficiency of medical services and brings tremendous economic benefits for patients and society. Besides, no drainage applied significantly reduced the postoperative pain score the next day after surgery from 1.89 ± 0.76 to 1.58 ± 0.63 (*P* = 0.022), but the pain score on discharge day between the two groups showed no difference (*p* > 0.05). These results are consistent with previous literature in COT that pain relief is evident on the first postoperative day (11, 32). A study using a small-sized drainage tube in endoscopic surgery showed no significant decrease in VAS pain score the next day after surgery (16), which could be attributed to insufficient size change. Our study made even more remarkable improvements by eliminating the drainage and demonstrating a significant reduction in postoperative pain, revealing the advantage of no drainage. We further discovered that the usage of pain medication in the no-drain group was less than that in the drain group. However, no significance was observed regarding the insufficient sample size. The improvements of reduced hospital stay and postoperative pain can boost the recovery of patients.

There are still several limitations in the present study that should be considered. It is a retrospective study with a relatively small sample size and still needs extensive data to clarify. A larger-scale prospective clinical trial is already being prepared in our center, with the goal of providing higher-level evidence in the future. As discussed above, this study excluded patients with potential risk factors for postoperative bleeding, and further exploration for these high-risk patients is needed. Besides, long-term follow-up including the cosmetic results, scar assessment, and patient satisfaction are still demanded. At last, for other endoscopic approaches like TOETVA, the safety of no drainage applied remains unknown, and we have already prepared a prospective clinical trial attempting to explore this issue (NCT04931576).

## Conclusion

In conclusion, we demonstrated that no drainage tube used during ETBAA does not increase the incidence of postoperative complications like hemorrhage, infection of surgical site, and subcutaneous seroma. In addition, this practice reduces the operation time, hospital stay, medical costs and postoperative suffering. No drainage tube applied has been shown to be safe and beneficial for the majority of patients who underwent ETBAA.

## Data Availability Statement

The raw data supporting the conclusions of this article will be made available by the authors, without undue reservation.

## Ethics Statement

The studies involving human participants were reviewed and approved by the Ethics Committee of the Second Affiliated Hospital, Zhejiang University School of Medicine (Ethic Approval No. 2021-0583). The patients/participants provided their written informed consent to participate in this study. Written informed consent was obtained from the individual(s) for the publication of any potentially identifiable images or data included in this article.

## Author Contributions

YC, JZ, and ZL contributed to the study conception and design. CW and YC searched prior articles and finished data analysis. BB and MY collected the clinical data. YC and JZ wrote the first draft of the manuscript. ZL revised the manuscript. All authors read and approved the final manuscript.

## Funding

This work was supported by the National Natural Science Foundation of China (81802334).

## Conflict of Interest

The authors declare that the research was conducted in the absence of any commercial or financial relationships that could be construed as a potential conflict of interest.

## Publisher's Note

All claims expressed in this article are solely those of the authors and do not necessarily represent those of their affiliated organizations, or those of the publisher, the editors and the reviewers. Any product that may be evaluated in this article, or claim that may be made by its manufacturer, is not guaranteed or endorsed by the publisher.
